# Influence of the Fukushima Daiichi Nuclear Power Plant Accident on the Use of Computed Tomography in Children With Mild Head Injuries

**DOI:** 10.2188/jea.JE20190158

**Published:** 2020-12-05

**Authors:** Shotaro Aso, Hiroki Matsui, Hideo Yasunaga

**Affiliations:** Department of Clinical Epidemiology and Health Economics, School of Public Health, The University of Tokyo, Tokyo, Japan

**Keywords:** radiation exposure, mild head injury, computed tomography, the Fukushima Daiichi Nuclear Power Plant accident, regression discontinuity

## Abstract

**Background:**

Computed tomography (CT) is commonly used in children with mild head injuries. People in Japan are concerned about radiation exposure and radiation-induced cancer because of the Fukushima Daiichi Nuclear Power Plant accident on March 11, 2011. This study investigated whether the accident influenced the use of CT in children with mild head injuries.

**Methods:**

Using the Japan Medical Data Center database, we identified patients aged ≤15 years visiting hospitals because of mild head injuries from January 1, 2008, to December 31, 2013. We excluded patients who were admitted to the hospital or received other medical examinations. Regression discontinuity analysis was used to compare proportions of patients undergoing head CT and having clinically important traumatic brain injury (ciTBI) overlooked before versus after the accident, adjusting for patient characteristics, secular trends, and hospital effect.

**Results:**

Eligible patients (*n* = 40,440) were classified as visiting the hospital before (*n* = 11,659) or after (*n* = 28,781) the accident. The regression discontinuity analysis showed that the accident was associated with a reduction in the proportion of patients undergoing head CT (odds ratio [OR] 0.73; 95% confidence interval [CI], 0.63–0.86), whereas the accident was not associated with an increase in cases where ciTBI was overlooked (OR 0.72; 95% CI, 0.13–4.00).

**Conclusions:**

The use of CT in children with mild head injuries declined after the Fukushima Daiichi Nuclear Power Plant accident. Improving awareness of radiation exposure risks among patients and physicians could reduce unnecessary CT.

## INTRODUCTION

Head injuries are common in children. Most of these injuries are mild and do not require head computed tomography (CT).^1^ Although CT is not invasive, it does carry a risk of radiation exposure, ranging from 15 to 30 millisieverts, and concern about radiation-induced cancer in children.^2^^–^^4^

To date, people in the United States and Japan seem tolerant toward medical radiation exposure. According to health data from the Organization for Economic Co-operation and Development, the number of CT examinations per person in the United States is highest all over the world, followed by Japan.^5^ A previous report showed that CT examination was routinely used in the United States, even in cases of mild head injury.^6^

However, an extremely severe disaster in Japan may have influenced people’s mindsets toward radiation exposure. The Great East Japan Earthquake and Tsunami hit the Fukushima Daiichi Nuclear Power Plant on March 11, 2011, resulting in nuclear meltdowns and the release of a large amount of radioactive material into the atmosphere.^7^ After the accident, correct and incorrect information about radiation exposure was widely disseminated in Japan by the mass media, and people in Japan became concerned about radiation exposure and radiation-induced cancer.^8^^,^^9^ Previous reports showed the percentages of people who were anxious about radiation exposure and its consequences were 71.6% in Fukushima and 40.4% outside of Fukushima,^8^ and telephone consultations about radiological examinations increased after the accident.^9^

We hypothesize that the occurrence of radiation leakage accidents may change people’s behavior regarding undergoing radiological examinations. To date, no studies have examined whether the Fukushima Daiichi Nuclear Power Plant accident and the related news coverage on radiation and radiation exposure reduced the use of radiological examinations.

Therefore, we conducted a retrospective cohort study using regression discontinuity analysis to investigate the association of the Fukushima Daiichi Nuclear Power Plant accident with the proportion of children with mild head injuries undergoing head CT examinations, using a multicenter outpatient and inpatient database in Japan.

## METHODS

Given the anonymous nature of the data, the requirement for informed consent was waived. The study was approved by the Institutional Review Board of the University of Tokyo (serial number: 10862-(1)).

### Data source

We used the Japan Medical Data Center database.^10^ The database includes health insurance claims data for approximately four million insured individuals in 2016. Most of these insured individuals are employees of Japanese companies and their families. The number of insured individuals participating the database increased year after year. The database includes administrative claims data for hospital visits and hospital admissions, with data on diagnoses, medical examinations, and treatments. Diagnoses were recorded based on International Classification of Disease 10th revision (ICD-10) codes,^11^ which are international classification codes used worldwide. Standard diagnostic names in accordance with the ICD-10 provided a code with a disease name in Japanese. This study used data from January 1, 2006, to December 31, 2015.

### Patient selection and outcome

We identified outpatients aged 15 years or younger who were diagnosed with superficial scalp injury (S00.0), contusion of eyelid and periocular area (S00.1), other superficial injuries of eyelid and periocular area (S00.2), multiple superficial head injuries (S00.7), concussion (S06.0), or unspecified head injury (S09.9). We also identified outpatients aged 15 years or younger who were diagnosed with suspected head injury (S01.0, 01.1, 02.0, 02.1, 02.7, 02.9, 06.2–9), because doctors in Japan record suspected diagnostic names when they implement any examination to diagnose a certain disease, even if the examination eventually produces negative results.

We excluded patients who were diagnosed with subcutaneous bleeding (S00.1), ecchymoma (S00.0), traumatic swelling (S00.2), incised wound (S01.0), chopping wound (S01.0), contused wound (S01.0), laceration (S01.0), or sting injury of the head (S01.1), based on standard diagnostic names in accordance with the ICD-10, because head CT is indicated for these conditions.^1^ We also excluded patients who were admitted to the hospital for head injury because their head injuries may not have been mild, as well as those who visited out-patient hospitals for head injuries in March 2011.

The primary outcome was undergoing CT. The secondary outcome was the having a clinically important traumatic brain injury (ciTBI) overlooked, which was defined as having a hospital admission caused by a ciTBI 1–14 days after the first medical examination for the head injury.

### Statistical analysis

We used a regression discontinuity design to analyze the association between the Fukushima Daiichi Nuclear Power Plant accident and the proportion of children undergoing CT, controlling for patient factors and possible temporal trends toward reduced proportions of patients undergoing CT during the study period.

Regression discontinuity designs can identify causal effects of interest in observational studies by exploring exogenous shifts in treatment probabilities, controlling for patient factors, and adjusting for preexisting temporal trends.^12^^–^^16^ In the regression discontinuity design, intervention is assigned to a subset of patients, based on a threshold of a baseline characteristic. The control group consists of a subset of patients below the threshold and the intervention group consists of patients above the threshold (ie, in this study, before and after the Fukushima Daiichi Nuclear Power Plant accident in March 2011). Due to the assignment rule, regression discontinuity design can achieve balance on unobserved factors, which mimics a randomized controlled trial. When estimating the treatment effect, a regression analysis compares treated to control patients, while adjusting for the assignment variable. Regression discontinuity provides a possible opportunity for obtaining unbiased causal effect estimates, when experiments are not feasible or when we want to evaluate interventions under “real-world” circumstances.^13^

To perform the regression discontinuity analysis, we estimated a patient-level logistic regression model to examine the association between the accident and the proportion of patients undergoing CT. A dummy variable, which played a role of assignment variable, was used to indicate whether the hospital visit was before or after the accident.^14^ We controlled for secular trends using variables for the year of the hospital visit.^15^ Patient baseline characteristics included age and sex. We use a logistic regression model with generalized estimating equations to control for clustering within hospitals.

We conducted a regression discontinuity analysis with a 6-year bandwidth from January 2008 to December 2013, with the month of the accident (March 2011) excluded as the main analysis. We also performed four additional regression discontinuity analyses with different bandwidths, including 2 years (from January 2010 to December 2011, with the month of the accident excluded), 4 years (from January 2009 to December 2012, with the month of the accident excluded), 8 years (from January 2007 to December 2014, with the month of the accident excluded), and 10 years (from January 2006 to December 2015, with the month of the accident excluded).

We calculated E-values as sensitivity analyses to assess the robustness of the results to potential residual or unmeasured confounders.^17^ The E-value is the minimum strength of association that unmeasured confounders would need to have with both the exposure and the outcome to explain away a treatment-outcome association. Rather than focusing on whether confounding of a specified strength would or would not suffice to explain away an effect estimate, E-values focus on the magnitude of the confounder associations that could produce confounding bias equal to the observed treatment-outcome association.

For the sensitivity analysis, we reanalyzed the data excluding contusion of eyelid and periocular area (S00.1), other superficial injuries of eyelid and periocular area (S00.2), and unspecified head injury (S09.9). Because the number of cases where ciTBI was overlooked was so small, we compared this number between groups using chi-square tests. We also changed the definition of ciTBI being overlooked, defining it as having a hospital admission caused by a ciTBI 1–7 days after the first medical examination for the head injury.

Differences in patient backgrounds before and after the accident were assessed using standardized differences. Standardized differences of less than 10% are considered negligible imbalances in baseline characteristics between the groups.^18^

Crude proportions of patients undergoing CT and having ciTBI overlooked before and after the accident were compared using chi-square tests or Fisher’s exact tests. A *P*-value less than 0.05 was considered to be significant. All statistical analyses were performed using Stata MP, Version 15.0 (Stata Corp, College Station, TX, USA).

## RESULTS

We identified 41,690 children who visited hospitals because of mild head injuries from 2008 to 2013. Of these patients, we excluded 774 who were admitted to the hospital and 476 who visited hospitals in March 2011. As a result, we selected 40,440 eligible patients, including 11,659 before the accident and 28,781 after the accident.

Table 1 shows patient baseline characteristics from 2008 to 2013 (patient baseline characteristics for other bandwidths are shown in eTable 1, eTable 2, eTable 3, and eTable 4). None of the baseline characteristics were different between patients visiting hospitals before and after the accident.

**Table 1.** Patient characteristics (2008–2013)

	Before the accident (*n* = 11,659)	After the accident (*n* = 28,781)	Standardized difference (%)
Age, *n* (%)			
0–6 months	728 (6.2)	2,121 (7.4)	4.5
7–18 months	2,208 (18.9)	5,376 (18.7)	0.7
19 months–3 years	2,180 (18.7)	5,125 (17.8)	2.3
3–6 years	2,134 (18.3)	5,214 (18.1)	0.5
7–12 years	3,148 (27.0)	7,438 (25.8)	2.6
13–15 years	1,261 (10.8)	3,507 (12.2)	4.3
Male, *n* (%)	7,146 (61.3)	17,613 (61.2)	0.2

The accident, The Fukushima Daiichi Nuclear Power Plant accident.

The crude proportion undergoing CT differed for patients visiting hospitals before versus after the accident (35.4% vs 33.2%, difference: −2.1%; 95% confidence interval [CI], −3.2 to −1.1). In seven cases, ciTBI was overlooked, with no difference before versus after the accident (0.02% [2/11,657] vs 0.02% [5/28,776]).

Table 2 shows the results of regression discontinuity analyses of undergoing CT, with different bandwidths. After controlling for patient characteristics, secular trends, and clustering within hospital, the accident was associated with a reduction in the proportion of patients undergoing CT from 2008 to 2013 (odds ratio [OR] 0.73; 95% CI, 0.63–0.86). Similar associations were seen in the analyses with different bandwidths.

**Table 2.** Regression discontinuity analyses comparing the odds of undergoing computed tomography before versus after the Fukushima Daiichi Nuclear Power Plant accident

Bandwidth for regression discontinuity	Odds ratio	95% confidence interval
2006–2015	0.56	0.48–0.65
2007–2014	0.67	0.57–0.78
2008–2013	0.73	0.63–0.86
2009–2012	0.81	0.69–0.94
2010–2011	0.80	0.69–0.94

Table 3 also shows the results of regression discontinuity analyses of ciTBI being overlooked, with different bandwidths. The accident was not associated with an increase in cases where ciTBI was overlooked from 2008 to 2013 (OR 0.72; 95% CI, 0.13–4.00). Similar associations were seen in the analyses with different bandwidths.

**Table 3.** Regression discontinuity analyses comparing the odds of clinically important traumatic brain injury being overlooked before vs after the Fukushima Daiichi Nuclear Power Plant accident

Bandwidth for regression discontinuity	Odds ratio	95% confidence interval
2006–2015	1.42	0.32–6.19
2007–2014	0.81	0.16–4.03
2008–2013	0.72	0.13–4.00
2009–2012	0.83	0.14–4.99
2010–2011	0.92	0.06–14.8

**Figure 1.**
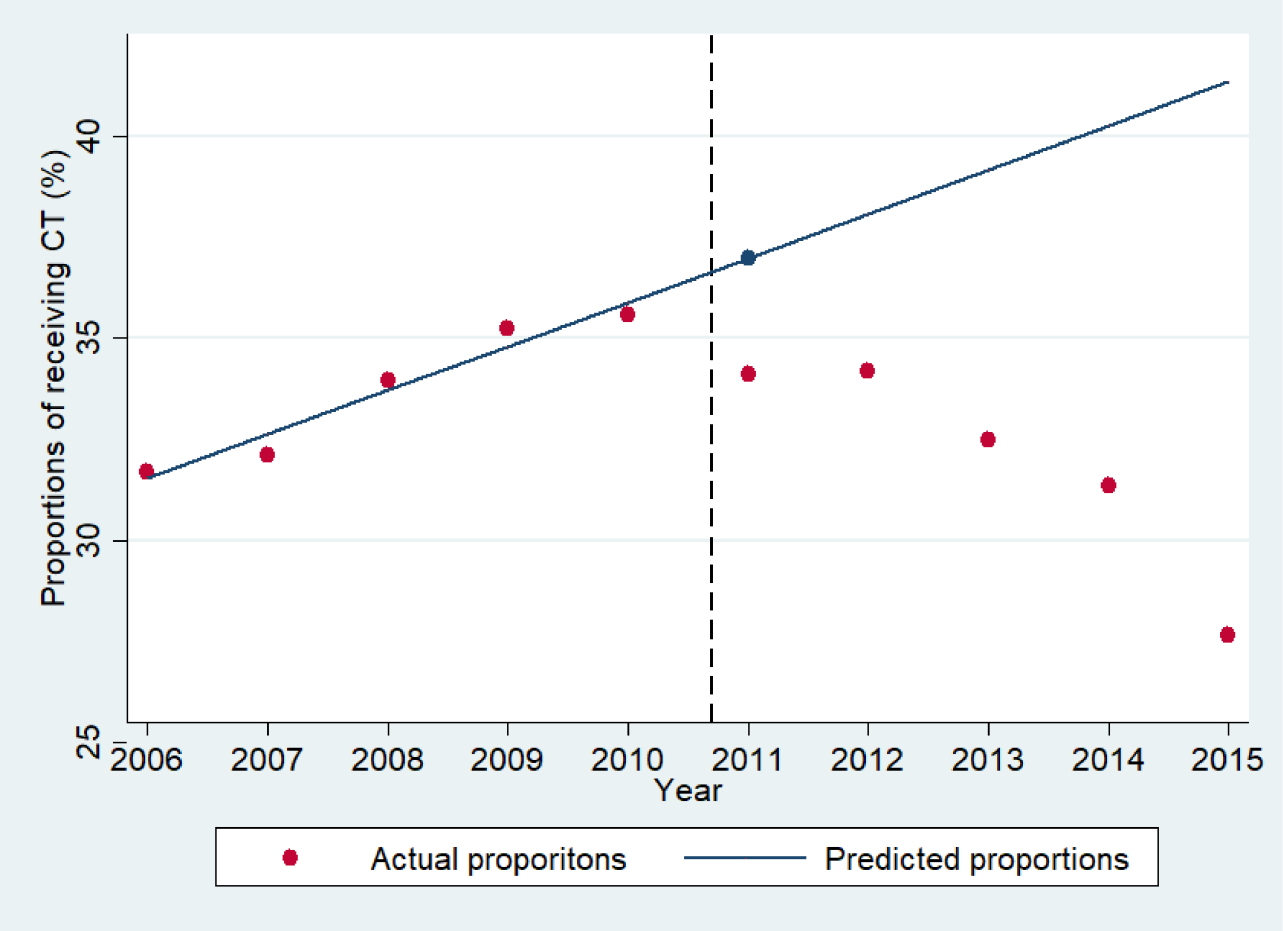
Trends in the proportions of children with mild head injuries undergoing CT examinations from 2006 to 2015. Red circles indicate the actual proportions of patients undergoing CT. The blue circle indicates the predicted proportion of patients undergoing CT in 2011. The blue line indicates the extrapolated prediction if the 2006–2010 trend in proportions of patients undergoing CT had continued. The black-dashed line indicates the month of the Fukushima Daiichi Nuclear Power Plant accident (March 2011).

Figure 1 plots the actual and predicted percentages of patients undergoing head CT. In 2011, the predicted percentage undergoing CT was 37.0%, and the actual percentage observed was 34.1%. After the accident, the actual percentage undergoing CT fell to 32.5% in 2013 and 27.7% in 2015. The E-value was 1.62, and the lower limit of the 95% CI closest to the null point was 1.37, with the bandwidth of 2008 to 2013.

In the sensitivity analysis, the accident was associated with a reduction in the proportion of patients undergoing CT from 2008 to 2013 (OR 0.77; 95% CI, 0.64–0.93). The percentages of cases where ciTBI was overlooked were 0.01% (1/8,602) and 0% (0/21,445) before and after the accident, respectively, and the accident was not associated with overlooked ciTBI (*P* = 0.114). The secondary analysis showed that one case of ciTBI was overlooked before the accident and that one case was overlooked after the accident. This analysis also showed that the accident was not associated with overlooked ciTBI (OR 0.35; 95% CI, 0.02–5.35).

## DISCUSSION

In this study, we estimated the causal effect of the Fukushima Daiichi Nuclear Power Plant accident followed by the Great East Japan Earthquake and Tsunami on undergoing head CT among children with mild head injuries using a multicenter outpatient and inpatient database in Japan. Our regression discontinuity analyses showed that the proportion of patients undergoing CT decreased after the Fukushima Daiichi Nuclear Power Plant accident, whereas cases of overlooking ciTBI did not increase after the accident.

Previous studies have identified indications for head CT for children with head injuries, such as altered mental status, history of vomiting, a dangerous mechanism of injury, and signs of basal skull fracture.^1^^,^^19^^,^^20^ One of these previous studies showed that the indications for performing head CT in children with head injuries partially depended on physicians’ experience and parental preferences, depending on the situation.^1^

Our results suggest that the Fukushima Daiichi Nuclear Power Plant accident reduced the behavior of performing CT in children. A potential reason for this is that people learned about the harms of radiation through the news coverage on radiation exposure and radiation-induced cancer. Before the accident, most people in Japan may have believed that the harm of their children undergoing CT was negligible, based on their low level of knowledge about the risk of radiation exposure, whereas they were anxious about their children’s injuries. Parents, therefore, expected physicians to order head CT for their children even when the injuries were mild. Although physicians understood that children with mild head injuries did not have to undergo head CT, physicians tended to adopt a “defensive” attitude to reduce their legal risks.

After the Fukushima Daiichi Nuclear Power Plant accident, there has been a great deal of news about radiation exposure, and most people in Japan have known about radiation exposure-induced cancer. Concern about radiation exposure has become commonplace. Parents of children with mild head injuries may avoid head CT, and physicians may have reconsidered the harmfulness of radiation exposure and may not recommend head CT to the parents.

Overlooked ciTBI did not increase after the accident. This may suggest that only unnecessary CT was reduced and that patients with moderate-to-severe head injuries may have continued to undergo CT as necessary.

Our results showed that the proportion of patients undergoing CT decreased each year after the Fukushima Daiichi Nuclear Power Plant accident. This may be because people in Japan have been continuously exposed to news about the accident. The problems related to the accident have not been solved, and the news about them has been regularly broadcast in Japan.^21^^,^^22^

This study had several strengths. The study was the first to show the association between the Fukushima Daiichi Nuclear Power Plant accident and a reduction in the use of CT using individual-level data. Although previous studies have suggested that there was a reduction in undergoing CT after the accident, these studies were based on aggregated data on a limited region of Fukushima Prefecture, which was near the plant, or in several children’s hospitals.^23^^,^^24^

A second strength was that the present study used regression discontinuity analysis, a quasi-experimental study design. Random variability implies that patients who visited hospitals before and after the accident will be similar on all observed and unobserved baseline characteristics, mimicking a randomized controlled trial.

Third, we used E-values for our sensitivity analyses. Observational designs generally preclude causal inference; that is, unmeasured confounders may persist despite adjustment for measured confounders. However, the E-value in our study was 1.62, and the lower limit of the 95% CI closest to the null point was 1.37 (>1), which indicates that considerable unmeasured confounding would be required to “explain away” the association between the accident and the use of CT.

Our findings are generalizable to all countries. CT scans for children with head injuries are widespread all over the world, and the Choosing Wisely Campaign has tried to reduce CT scans for these children.^25^

There were also several limitations to this study. The database did not include detailed patient data, such as vomiting, loss of consciousness, mental status, and the mechanism of injury. However, we excluded patients who were hospitalized because of head injury. Second, our study population did not completely reflect the population of Japan as a whole, because the database included only employees of Japanese companies with health insurance and their families and did not include unemployed adults. Third, the safety of reducing CT scans could not be validated because there were few cases of ciTBI being overlooked. It is possible that there were few such cases because we identified only patients with mild head injuries and ciTBI may be inherently lower in this group.

In this study, a regression discontinuity design using a health insurance claims database showed that the proportion of children with mild head injuries undergoing head CT declined after the Fukushima Daiichi Nuclear Power Plant accident. Improving awareness of radiation exposure risks among patients and physicians could reduce unnecessary CT.

## APPENDIX A. SUPPLEMENTARY DATA

eTable 1. Patient characteristics (2010–2011)

eTable 2. Patient characteristics (2009–2012)

eTable 3. Patient characteristics (2007–2014)

eTable 4. Patient characteristics (2006–2015)

## References

[1] Kuppermann N, Holmes JF, Dayan PS, Identification of children at very low risk of clinically-important brain injuries after head trauma: a prospective cohort study. Lancet. 2009;374(9696):1160–1170. 10.1016/S0140-6736(09)61558-0 19758692

[2] Parker L Computed tomography scanning in children: radiation risks. Pediatr Hematol Oncol. 2001;18(5):307–308. 10.1080/088800101300312564 11452401

[3] Pearce MS, Salotti JA, Little MP, Radiation exposure from CT scans in childhood and subsequent risk of leukaemia and brain tumours: a retrospective cohort study. Lancet. 2012;380:499–505. 10.1016/S0140-6736(12)60815-0 22681860 PMC3418594

[4] Berrington de González A, Mahesh M, Kim KP, Projected cancer risks from computed tomographic scans performed in the United States in 2007. Arch Intern Med. 2009;169(22):2071–2077. 10.1001/archinternmed.2009.440 20008689 PMC6276814

[5] Health at a Glance 2017. doi:10.1787/health_glance-2017-en. 10.1787/health_glance-2017-en

[6] Burstein B, Upton JEM, Terra HF, Neuman MI Use of CT for head trauma: 2007–2015. Pediatrics. 2018;142(4):e20180814. 10.1542/peds.2018-0814 30181120

[7] The National Diet of Japan The Fukushima Nuclear Accident Independent Investigation Commission. http://warp.da.ndl.go.jp/info:ndljp/pid/3856371/naiic.go.jp/wp-content/uploads/2012/09/NAIIC_report_hi_res10.pdf. Accessed June 20, 2018.

[8] Okazaki R, Ootsuyama A, Abe T, Kuto T A questionnaire survey about public’s image of radiation after the Fukushima Daiichi nuclear power plant accident. J UOEH. 2012;34(1):91–105. 10.7888/juoeh.34.91 22428463

[9] Kanda R, Tsuji S, Eguchi-Kasai K, Yonehara H, Torikoshi M Telephone consultations on radiation exposure: tabulated results from the year following the TEPCO Fukushima Daiichi Nuclear Power Station accident. Annu Rep Natl Inst Radiol Sci. 2014;(52):112–113. http://search.jamas.or.jp/link/ui/2015050736.

[10] Kimura S, Sato T, Ikeda S, Noda M, Nakayama T Development of a database of health insurance claims: standardization of disease classifications and anonymous record linkage. J Epidemiol. 2010;20(5):413–419. 10.2188/jea.JE20090066 20699602 PMC3900837

[11] WHO Classification of Diseases. WHO. https://www.who.int/classifications/icd/en/. Published 2019. Accessed September 4, 2019.

[12] Bor J, Moscoe E, Mutevedzi P, Newell ML, Bärnighausen T Regression discontinuity designs in epidemiology: causal inference without randomized trials. Epidemiology. 2014;25(5):729–737. 10.1097/EDE.0000000000000138 25061922 PMC4162343

[13] Van Leeuwen N, Lingsma HF, de Craen AJM, Regression discontinuity design: simulation and application in two cardiovascular trials with continuous outcomes. Epidemiology. 2016;27(4):503–511. 10.1097/EDE.0000000000000486 27031038

[14] Glance LG, Osler TM, Mukamel DB, Meredith JW, Dick AW Effectiveness of nonpublic report cards for reducing trauma mortality. JAMA Surg. 2014;149(2):137–143. 10.1001/jamasurg.2013.3977 24336907

[15] Melamed A, Fink G, Wright AA, Effect of adoption of neoadjuvant chemotherapy for advanced ovarian cancer on all cause mortality: quasi-experimental study. BMJ. 2018;360:j5463. 10.1136/bmj.j5463 29298771 PMC5751831

[16] Imbens GW, Lemieux T Regression discontinuity designs: a guide to practice. J Econom. 2008;142(2):615–635. 10.1016/j.jeconom.2007.05.001

[17] VanderWeele TJ, Ding P Sensitivity analysis in observational research: introducing the E-value. Ann Intern Med. 2017;167(4):268–274. 10.7326/M16-2607 28693043

[18] Austin PC Balance diagnostics for comparing the distribution of baselinecovariates between treatment groups in propensity-scorematched samples. Stat Med. 2009;28:3083–3107. 10.1002/sim.3697 19757444 PMC3472075

[19] Dunning J, Daly JP, Lomas JP, Derivation of the children’s head injury algorithm for the prediction of important clinical events decision rule for head injury in children. Arch Dis Child. 2006;91(11):885–891. 10.1136/adc.2005.083980 17056862 PMC2082967

[20] Osmond MH, Klassen TP, Wells GA, CATCH: a clinical decision rule for the use of computed tomography in children with minor head injury. CMAJ. 2010;182(4):341–348. 10.1503/cmaj.091421 20142371 PMC2831681

[21] Fukushima Daiichi Decommissioning Project | Tokyo Electric Power Company Holdings. https://www7.tepco.co.jp/responsibility/decommissioning/action/index-e.html. Accessed August 25, 2018.

[22] Mid-and-Long-Term Roadmap towards the Decommissioning of TEPCO’s Fukushima Daiichi Nuclear Power Station. http://www.meti.go.jp/english/earthquake/nuclear/decommissioning/pdf/20170926_01a.pdf. Accessed August 25, 2018.

[23] Yoshida K, Hayashida N, Fukushima Y, Changes in radiological imaging frequencies in children before and after the accident at the Fukushima Daiichi Nuclear Power Plant in Fukushima Prefecture, Japan. Jpn J Radiol. 2015;33(10):619–626. 10.1007/s11604-015-0464-8 26219903

[24] Miyazaki O Has pediatric CT practice in Japan changed since the Fukushima nuclear disaster? Pediatr Radiol. 2015;45(10):1571–1574. 10.1007/s00247-015-3295-y 25792153

[25] CT Scans for Children with Head Injuries Choosing Wisely. http://www.choosingwisely.org/patient-resources/ct-scans-for-children-with-head-injuries/. Accessed June 20, 2018.

