# Supplementary material for: Influence of the Fukushima Daiichi Nuclear Power Plant Accident on the Use of Computed Tomography in Children With Mild Head Injuries
Source: J Epidemiol. 2020 Dec 5;30(12):542–6. doi: 10.2188/jea.JE20190158 (PMC7661337; doi:10.2188/jea.JE20190158)
Supplement: Supplementary file 1 [file je-30-542-s001.pdf]

eTable 1. Patient characteristics (2010–2011)

	Before the accident (n=6,237)		After the accident (n=5,890)		Standardized difference (%)
Age, n (%)					
0–6 months	384	(6.2)	434	(7.4)	4.8
7–18 months	1,164	(18.7)	1,144	(19.4)	1.9
19 months–3 years	1,154	(18.5)	1,067	(18.1)	1.0
3–6 years	1,153	(18.5)	1,087	(18.5)	0.1
7–12 years	1,713	(27.5)	1,465	(24.9)	5.9
13–15 years	669	(10.7)	693	(11.8)	3.3
Male, n (%)	3,878	(62.2)	3,623	(61.5)	1.4

eTable 2. Patient characteristics (2009–2012)

	Before the accident (n=15,014)		After the accident (n=24,504)		Standardized difference (%)
Age, n (%)					
0–6 months	569	(6.1)	1,092	(7.2)	3.4
7–18 months	1,747	(18.8)	2,855	(18.8)	0.0
19 months–3 years	1,719	(18.5)	2,732	(18.0)	0.9
3–6 years	1,693	(18.2)	2,754	(18.2)	0.1
7–12 years	2,538	(27.3)	3,855	(25.4)	3.2
13–15 years	1,031	(11.1)	1,864	(12.3)	2.9
Male, n (%)	5,717	(61.5)	9,352	(61.7)	0.2

eTable 3. Patient characteristics (2007–2014)

	Before the accident (n=13,406)		After the accident (n=42,984)		Standardized difference (%)
Age, n (%)					
0–6 months	859	(6.4)	3,193	(7.4)	4.0
7–18 months	2,564	(19.1)	7,990	(18.6)	1.4
19 months–3 years	2,495	(18.6)	7,646	(17.8)	2.1
3–6 years	2,471	(18.4)	7,646	(17.8)	1.7
7–12 years	3,609	(26.9)	11,228	(26.1)	1.8
13–15 years	1,408	(10.5)	5,281	(12.3)	5.6
Male, n (%)	8,192	(61.1)	26,341	(61.3)	0.4

eTable 4. Patient characteristics (2006–2015)

	Before the accident (n=15,154)		After the accident (n=56,819)		Standardized difference (%)
Age, n (%)					
0–6 months	975	(6.4)	4,329	(7.6)	4.6
7–18 months	2,847	(18.8)	10,747	(18.9)	0.3
19 months–3 years	2,838	(18.7)	10,086	(17.8)	2.5
3–6 years	2,878	(19.0)	9,872	(17.4)	4.2
7–12 years	4,039	(26.7)	14,761	(26.0)	1.5
13–15 years	1,577	(10.4)	7,024	(12.4)	6.2
Male, n (%)	9,269	(61.2)	34,775	(61.2)	0.1
